# HIV-1 Nef: a multifaceted modulator of T cell receptor signaling

**DOI:** 10.1186/1478-811X-10-39

**Published:** 2012-12-10

**Authors:** Libin Abraham, Oliver T Fackler

**Affiliations:** 1 Department of Infectious Diseases, Virology, University Hospital Heidelberg, INF 324, Heidelberg, 69120, Germany; 2 The Hartmut Hoffmann-Berling International Graduate School of Molecular and Cellular Biology, INF 501, Heidelberg, 69120, Germany

**Keywords:** HIV/AIDS, Nef, T-cell receptor signaling, Microclusters

## Abstract

Nef, an accessory protein of the Human Immunodeficiency Virus type 1 (HIV-1), is dispensable for viral replication in cell culture, but promotes virus replication and pathogenesis in the infected host. Acting as protein-interaction adaptor, HIV-1 Nef modulates numerous target cell activities including cell surface receptor expression, cytoskeletal remodeling, vesicular transport, and signal transduction. In infected T-lymphocytes, altering T-cell antigen receptor (TCR) signaling has long been recognized as one key function of the viral protein. However, reported effects of Nef range from inhibition to activation of this cascade. Recent advances in the field begin to explain these seemingly contradictory observations and suggest that Nef alters intracellular trafficking of TCR proximal machinery to disrupt plasma membrane bound TCR signaling while at the same time, the viral protein induces localized signal transduction at the trans-Golgi network. This review summarizes these new findings on how HIV-1 Nef reprograms TCR signalling output from a broad response to selective activation of the RAS-Erk pathway. We also discuss the implications of these alterations in the context of HIV-1 infection and in light of current concepts of TCR signal transduction.

## Background

### T-cell receptor signaling

Development, proliferation and immune functions of T-lymphocytes are regulated by their activation state [1]. In concert with co-stimulatory signals, T-cell activation is primarily governed by engagement of surface exposed T-Cell Antigen Receptor (TCR) complexes with Major Histocompatibility Complex (MHC) bound peptides on antigen-presenting cells (APC). These interactions occur physiologically in the context of stable cell-cell contacts referred to as immunological synapse (IS) and trigger a broad range of downstream signaling events including sequential tyrosine phosphorylation cascades, rapid elevation of intracellular calcium flux and dynamic F-actin remodeling [2-11]. These plasma membrane–associated and cytoplasmic events are transmitted to the nucleus by activation and/or import of transcription factors that launch specific transcriptional profiles characteristic for activated T-cells, including induced expression of the T-cell survival cytokine interleukin 2 (IL-2).

**Figure 1:**
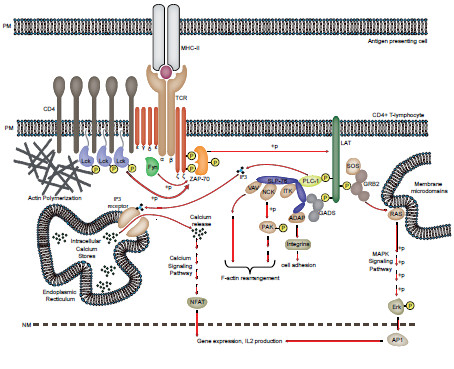
**Schematic overview of TCR signaling.** Simplified scheme of TCR signal transduction and effector functions. Following TCR ligation with peptide-loaded MHC-class II molecules, F-actin is polymerized at plasma membrane sites of TCR engagement. ITAMs of TCR zeta chains are phosphorylated by the SRC family kinases Lck and Fyn to recruit the downstream kinase ZAP-70. ZAP-70 is activated by phosphorylation, primarily by Lck but also by Fyn, and active ZAP-70 phosphorylates the membrane adaptor LAT at multiple residues. Phospho-LAT serves as scaffold for macromolecular signaling assemblies, via multiple interactions including with PLCγ1, SLP-76 via GADS, and SOS via GRB2. The cytoplasmic adaptor SLP-76 binds to multiple proteins including VAV, NCK, ITK, PLCγ1 and ADAP to regulate signaling pathways necessary for F-actin rearrangement and cell adhesion. PLCγ1 catalyzes formation of IP3 and DAG from PIP2 (not shown). The soluble secondary messenger IP3 binds to IP3R to induce calcium release from intracellular stores and trigger NFAT activation. Active LAT also activates RAS in membrane microdomains via GRB2-SOS which triggers MAPK signaling, culminating in phosphorylation and activation of Erk. Nuclear translocation of activated NFAT/Erk induces gene transcription and IL2 production, hallmarked by T-cell proliferation and differentiation. GRB2: Growth factor receptor-bound protein 2, SOS: son of sevenless, Erk: extracellular-signal-regulated kinase, AP1: Activator protein-1, IP3: Inositol 1,4,5-trisphosphate, CDC42: cell division cycle 42, WAVE2: WASP-family verprolin-homologous protein-2, WASP: Wiskott Aldrich syndrome protein, PAK: p21 activated protein kinase, PM: plasma membrane, NM: nuclear membrane. ‘+p’ and yellow circles with ‘P’ indicate phosphorylation events and phosphorylation, respectively.

TCR signaling in CD4^+^ T-cells is initiated by the interaction of the TCR α,β subunits with peptide-loaded MHC-II causing spatial rearrangements of the multi-subunit TCR complex. As one result of peptide loading, the cytoplasmic TCR zeta chain undergoes conformational changes to expose immunoreceptor tyrosine-based activation motifs (ITAMs), which become subsequently phosphorylated by the Src family kinase Lck (lymphocyte-specific protein tyrosine kinase) [12]. Phospho-ITAMs recruit the downstream kinase ZAP-70 (zeta chain associated protein of 70 kDa), which is also phosphorylated and activated by Lck [10,13,14]. Active ZAP-70 now initiates a cascade of phosphorylation events, with the most important ZAP-70 phosphorylation substrates being the trans-membrane adapter protein LAT (linker for the activation of T-cells) and the cytosolic adapter protein SLP-76 (Src homology 2 (SH2) domain–containing leukocyte phosphoprotein of 76 kD) [11,13,15-18]. These two adapters form the backbone of a signaling complex that organizes an array of effector molecules in the correct spatiotemporal manner to allow for the activation of multiple downstream signaling pathways. These include PLCγ1 (phospholipase Cγ1) that controls intracellular Ca^(2+)^ flux, F-actin remodeling, cell adhesion and activation of the MAPK pathway, that all together synergize to optimal production of IL-2 by activation of transcription factors such as NFAT (nuclear factor of activated T-cells) and AP-1 (activator protein-1) [11,13,17,18]. Thus, signals emanating from Lck trigger TCR proximal signal transduction and are diversified and channeled to multiple downstream signaling pathways by LAT-SLP-76 adaptor scaffolds that act as mirco-signalosomes (Figure 1) [17,18]. In this scenario, individual signal transduction steps are spatially separated: TCR engagement triggers the segregation of initial signaling modules including TCR, Lck and ZAP-70 (‘primary signaling domains’) from LAT-SLP-76 signaling scaffolds that are considered ‘secondary signaling domains’ or ‘signal diversification and regulation modules’ [13,19,20]. Subsequent TCR distal events are compartmentalized due to the localization of RAS signaling to Golgi membranes [21-28].

### Organization of TCR proximal signaling microclusters

This physical segregation of key TCR proximal signaling molecules can be visualized at the IS by virtue of their organization in distinct supra molecular activation clusters (SMAC) that, depending on their position relative to the IS, are referred to as central, peripheral and distal SMAC [2,5,29,30]. Providing T-lymphocytes with planar surface-bound TCR stimulation in the form of lipid bilayers or coated glass surfaces allows to detect the formation of proximal TCR signal transduction units referred to as microclusters (MCs) [31]. MCs are formed within seconds after initial TCR engagement by clustering of the TCR complex itself and various TCR proximal signaling molecules, range from 30-300nm in size [32], and are considered ‘signaling hotspots’ essential for T-cell activation [29,32-35]. The most widely studied MCs are those nucleated around the TCR itself (TCR MCs) and around the adaptor proteins LAT and SLP-76 (LAT/SLP-76 MCs). It is well established that activation of T-cell requires the formation of these structures [31,36,37]. Both actin dynamics and microtubule-mediated vesicular transport play important roles in orchestrating the induction and dynamic movement of signaling MCs [5,10,11,30,38-40], subsequently leading to downstream signaling and expression of target genes. Proximal TCR signaling can thus be viewed as a consequence of highly coordinated interactions of individual signaling modules with specific composition and spatio-temporal regulation.

### Coupling of HIV-1 replication to T-cell activation

HIV-1, the causative agent of the Acquired Immunodeficiency Syndrome, AIDS, infects target cells that present the entry receptors and co-receptors CD4 and CXCR4/CCR5 on their surface. This receptor specificity determines the tropism of HIV-1 for CD4^+^ T-lymphocytes and monocytes/macrophages that constitute the main target cells of the virus in its human host. In particular in CD4^+^ T-lymphocytes, the efficacy of HIV-1 replication is tightly coupled to the activation state of these target cells: while HIV-1 readily undergoes multiple rounds of replication in activated memory CD4^+^ T-cells, resting helper T-cells are refractory to productive infection [41-46]. Blocks to HIV-1 infection in resting CD4^+^ T-lymphocytes include the entry step, completion of reverse transcription (RT) of incoming viral RNA genomes into DNA, nuclear import and integration of the viral genome, as well as transcription of viral genes [45-49]. The barrier to viral entry is posed by the rigid cortical actin layer and HIV-1 triggers specific signaling cascades to bypass this obstacle, explaining why HIV-1 entry is readily observed in resting CD4^+^ T-lymphocytes [50]. The block to reverse transcription is controlled by the recently identified host cell restriction factor Samhd1 [51-53]. This Samhd1 dependent restriction might reflect the ability of this enzyme to reduce the pool of available dNTPs required for RT in resting but not activated T-lymphocytes, the latter of which display significantly higher dNTP levels. Inefficient transcription of viral genes in resting T-cells finally mirrors the low abundance/activity of host cell transcription factors such as nuclear factor-κB (NF-κB) and NFAT required for initial rounds of viral transcription prior to synthesis of the viral transcription factor Tat [49,54-57]. Together, this implies that the activation state of target CD4^+^ T-lymphocytes not only dictates the success rate of HIV-1 infection of previously uninfected target cells, but also determines how efficiently integrated proviral genes are transcribed, and thus new virus particles are synthesized in latently infected resting cells upon encounter with T-cell stimuli. Of note, activation of full rounds of HIV-1 replication in resting T-cells can be achieved by inducing yet ill-defined, intermediate levels of T-cell activation [58]. This preference of HIV-1 for target T-lymphocytes with intermediate activation states may reflect that potent activation through TCR ligation can trigger activation induced cell death (AICD) [59,60] which would significantly reduce the life span of productively infected CD4^+^ T-cells and may thus self-limit spread of HIV-1 infection. It is thus plausible that HIV-1 applies strategies to fine tune T-cell activation in infected cells in order to optimize its replication. The viral protein Nef emerges as a central viral player in this scenario and this review will summarize recent advances in our understanding on the mechanisms employed by Nef.

### The Nef protein and its effects on TCR signaling

Nef is a small 27 – 35 kDa myristoylated protein encoded the primate lentiviruses (HIV-1, HIV-2 and SIV) that localizes to the cytoplasm of infected cells and is partially recruited to cellular membranes. Nef is dispensable for HIV-1 replication in cell culture, however in the infected host, the viral protein markedly elevates virus titers and is required for rapid disease progression [61-63]. Nef is therefore considered a key factor in AIDS pathogenesis [44,64]. It is generally assumed that Nef exerts this role in AIDS pathogenesis via several independent activities that (i) directly promote HIV-1 replication and (ii) facilitate evasion of infected cells from recognition by the host immune system (see [64,65] for reviews). These activities are mediated by a plethora of suggested interactions with host cell proteins, by which Nef manipulates various aspects of host cell vesicular transport, cytoskeleton dynamics and cell motility, as well as signal transduction [44,64,66,67]. In the context of CD4^+^ T lymphocytes, most studies since the early days of Nef characterization focused on effects of Nef on T-cell receptor signaling.

The most impressive evidence supporting a direct impact of Nef on T-lymphocyte activation was obtained in macaques infected with the acutely lethal SIV variant pbj14, in which T-lymphocyte hyperactivation and subsequent depletion was observed [68]. This effect reflected the ability of this particular Nef variant to trigger T-cell receptor signaling and IL-2 production by virtue of an ITAM motif not present in any other Nef protein [68-70]. Even though significantly milder than Nef from SIVpbj14, HIV-1 Nef was also demonstrated to bear the capacity to enhance basal levels of T-cell activation, especially when physically tethered to the plasma membrane (PM) [71]. These initial observation initiated numerous studies to address extend, mechanism and functional consequence of alterations of TCR signaling by HIV-1 Nef. Presumably due to the use of varying experimental strategies that often involved the use of immortalized cell lines rather than primary cells and marked overexpression of individual proteins, these efforts yielded sometimes contradicting results but generally concluded that HIV-1 Nef inhibits T-cell activation [72-75]. More recent work, including studies based on viral infection of primary target T-cells, revealed that HIV-1 Nef moderately enhances distal responses to exogenous TCR stimulation by mitogens or anti-TCR Abs including enhanced induction of NFAT, NFκB, AP-1 transcriptional activity, the release of calcium, and IL-2 production [50,76-88]. Taken together these studies provided evidence that HIV-1 Nef can lower the threshold for activation of CD4^+^ T-lymphocytes, but is not sufficient to cause activation in the absence of exogenous stimuli.

Many aspects of HIV-1 Nef induced TCR signaling dysfunction are also observed with Nef proteins from simian immunodeficiency viruses (SIV) [89-92] which, in contrast to HIV-1, are largely non-pathogenic in their natural host. In addition to the effects exerted by HIV-1 Nef, SIV Nef proteins also efficiently downregulate CD3-TCR complexes from the surface of infected T lymphocytes and thus more potently inhibit TCR signaling [83,92-95]. This differential regulation of TCR signaling by HIV-1 and SIV Nef proteins has been suggested as one determinant of the pathogenic outcome of HIV-1 infection in humans [83,92]. Since molecular and functional differences between SIV and HIV-1 Nef proteins in this context were already discussed extensively (see reviews [44,64,96,97]), this review focuses on recent developments that enhance our understanding of how HIV-1 Nef modulates TCR signaling in infected CD4^+^ T-lymphocytes.

The concept that HIV-1 Nef slightly enhances TCR signaling was at odds with another series of studies demonstrating rather pronounced inhibitory effects of Nef on formation and organization of the IS and thus early events following TCR engagement. One prominent consequence of HIV-1 infection or isolated expression of Nef is pronounced accumulation of the TCR itself and the proximal kinase Lck in an intracellular compartment [89,90,98,99]. Based on these findings, Nef interferes with essential hallmarks of TCR signal initiation, leaving the field with the paradoxal situation that Nef facilitates some aspects of distal TCR signaling while early events in the TCR cascade are potently inhibited by the viral protein. Recent studies now started to provide more detailed insight into the mechanisms underlying these seemingly contradictory effects of Nef, allowing depiction of an integrative view on how Nef rewires TCR signaling by affecting both, TCR proximal and distal events.

### Decompartmentalization of TCR signaling by HIV-1 Nef

**Figure 2:**
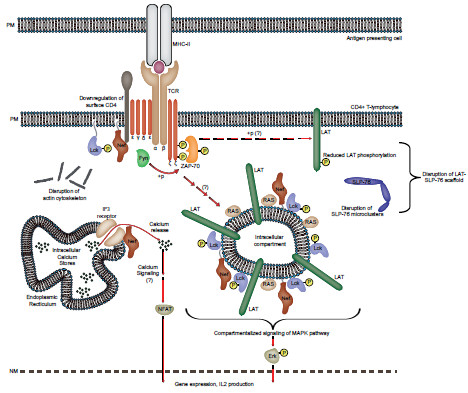
**Model of the effects of HIV-1 Nef on TCR signaling.** Schematic representation of TCR signaling in presence of HIV-1 Nef. Expression of Nef down regulates cell surface exposure of the CD4 receptor. In addition, Nef strongly reduces the availability of Lck, LAT and active RAS at the plasma membrane as these components are retargeted to an intracellular compartment in their active form. This compartment represents the TGN for Lck and RAS and it is assumed here that LAT is recruited to the same compartment. Nef also localizes to these membranes; however, whether its physical presence is required for TCR rewiring remains unclear. Upon TCR stimulation, triggered F-actin remodeling is inhibited by Nef by virtue of its interaction with PAK2 (not shown). The SRC kinase Fyn, whose plasma membrane localization is unaffected by Nef, phosphorylates TCR zeta and ZAP-70, however ZAP-70 mediated phosphorylation of LAT and subsequent SLP-76 microcluster formation is potently disrupted by Nef. Presumably again through the activity of Fyn (indicated by the dotted arrow and question mark), TCR engagement also stimulates Lck-RAS at intracellular membranes, generating MAPK signaling resulting in the activation of Erk. In addition, Nef binds to IP3 receptor to induce calcium release and NFAT activation. By inducing a constitutively active intracellular Lck-RAS signaling module that is partially uncoupled from the plasma membrane, Nef tailors a narrow TCR downstream response that likely optimizes HIV-1 spread in the infected host.

The finding that Nef retargets Lck, the master switch kinase of early TCR signaling, away from the plasma membrane to early and recycling endosomes (RE) as well as the trans-Golgi network (TGN) provided a first important clue [98,100]. Since TCR signaling is initiated and sustained at the PM and Lck is essential to this process, this finding was entirely consistent with the disruption of early TCR signaling observed in Nef expressing CD4^+^ T-lymphocytes. However, only with the recent determination of the subcellular localization of kinase-active Lck, it became evident that this retargeting might also be linked to the ability of Nef to enhance distal aspects of TCR signaling [100]. The use of phospho-specific antibodies revealed that RE/TGN associated Lck subpopulations in Nef expressing cells are in the catalytically active conformation and thus signaling competent. Of note, this intracellular enrichment of active Lck kinase was observed already in the absence of exogenous TCR stimulation, indicating that Nef may generate constitutive intracellular signals. While the majority of early TCR signaling occurs at the plasma membrane, compartmentalization of the pathway has been described in the activation of the RAS GTPase, which takes place at intracellular membranes, including the Golgi apparatus [21-28]. Similar to activation of the phospho-tyrosine cascade at the PM, also RAS activation depends on prior induction of Lck [27]. Consistently, the enrichment of active Lck at RE/TGN compartments induced by Nef resulted in an increase of localized RAS activity and enhanced activation of Erk kinase as well as IL-2 production downstream of RAS in response to exogenous stimulation [100] (see Figure 2). This effect may synergize to potentiate transcriptional activation of downstream target genes with the ability of Nef to (i) promote, via interactions with the IP3 receptor, the release of calcium from intracellular stores [81] and (ii) trigger Erk activity via association with the Nef-associated kinase complex NAKC [101-103].

Together, these findings provide an explanation for the observed Nef-mediated disruption of early TCR signaling and selective, stimulus-independent sensitization of the RAS pathway: by aggregating a specific set of signaling competent TCR effectors away from the PM to the TGN, Nef tailors TCR responses from a typically broad cascade of effectors to a specific activation of the RAS pathway. However, the effect of Nef on signaling downstream of RAS becomes only apparent following TCR engagement, raising the question how such signal transmission might occur in the absence of Lck from the PM. It has long been noted that the pivotal role of Lck in proximal TCR signaling can be substituted by the closely related Src kinase Fyn, however, breadth and magnitude of this functional redundancy has remained controversial [12,104-108]. Interestingly and in sharp contrast to Lck, the localization of active Fyn was unaltered in Nef expressing cells prior and following TCR stimulation [100]. Selective interference of Nef with PM localization of Lck but not of Fyn may account for the residual responsiveness of Nef-expressing or HIV-1 infected cells to TCR stimulation.

### Disruption of proximal TCR events

Recent studies also provided surprising insights into the complexity of mechanisms employed by HIV-1 Nef to disrupt early TCR signaling events. Earlier work had already established that Nef interferes with actin remodeling and cell spreading triggered by TCR engagement [89,90,99]. On the molecular level, Nef controls actin dynamics by virtue of its association with the cellular kinase PAK2, which is turned by Nef to phosphorylate and thus inactivate the actin severing factor cofilin to reduce actin remodeling [91,109]. It however became increasingly clear that this actin-mediated mechanism may be necessary but not sufficient for the observed disruption of early TCR signaling. Taking advantage of the TCR microcluster concept for visualization and quantification of effects of Nef on early TCR signaling allowed the identification of the specific steps affected by Nef. Again possibly reflecting the action of Fyn and in line with earlier reports [89,92], Nef had no effect on the extend of ZAP-70 activation or its organization in signaling microclusters [110]. ZAP-70 activation therefore does not require cell spreading and dynamic actin remodeling. Pronounced defects, however, were observed in the presence of Nef for the organization of the adaptor protein SLP-76 in microcluster. As a consequence, physical association between SLP-76 and LAT, a key event in TCR signal transmission, was drastically reduced by the viral protein. Interestingly, further mechanistic analysis revealed this effect to be mediated by Nef via simultaneous interference with actin remodeling/cell spreading and disruption of the subcellular localization of LAT. Very similar to the effect on Lck, Nef induced the accumulation of LAT in intracellular compartments [110]. Nef mediated retargeting of both proteins can be overcome by coexpression of the anterograde transport adaptor Unc119 [100,110]. How Unc119, which is physiologically involved in the anterograde transport of Lck by virtue of its interaction with the GTPase Rab11 [111], acts to overwrite the Nef-mediated block and whether this reflects the involvement of endogenous Unc119 in the inhibitory action of Nef remains to be determined.

### Functional consequences of Nef-mediated alterations of TCR signaling

The fact that evolution selected for several mechanisms by which HIV-1 Nef synergistically tunes TCR signaling suggests that this provides HIV with a decisive advantage in the infected host. It is well established that effects of Nef on HIV-1 replication in T-lymphocyte cultures are most pronounced when unstimulated cells are infected and only subsequently subjected to TCR stimulation [112,113]. This positive effect on HIV-1 replication appears to correlate with the ability of Nef to induce RE/TGN-associated signaling [100]. This may reflect e.g. that activation induced cell death of Nef-expressing cells following TCR stimulation is slightly reduced relative to non-Nef expressing control cells and that Nef-tailored TCR signaling could promote HIV-1 transcription [83,114]. However, the Nef effect on virus replication in this cell system is modest and in magnitude significantly lower than that observed in e.g. SIV infected rhesus macaques [63]. Its relevance in the context of an immunocompetent host is hence unclear. It appears thus plausible that Nef-tailored TCR responses bear consequences that affect HIV-1 spread and pathogenesis beyond its direct replicative capacity. Nef has been reported to trigger the release of exosome microvesicles that are massively secreted to bystander cells upon T-cell activation [115,116]. Enhanced exosome release was directly linked to activation of Erk signaling via the association of Nef with a large cellular kinase complex referred to as NAKC [101,103,117]. Mechanisms of decompartmentalization of TCR signaling and Erk activation may thus integrate to modulate exosome release in response to TCR activation, with the Erk-RAS pathway emerging as the central target of Nef action. Depending on their composition and cargo, such exosomes could possibly render non-infected bystander cells more susceptible for infection or undermine potent immune recognition of already infected cells. On the other hand, Nef has been implicated as a major determinant of killing of non-infected CD4^+^ T-lymphocytes in HIV-1 infection of tonsil histocultures [118-120] and, much more prominently, in humanized mice [121]. Since Nef-induced exosomes are able to induce such bystander T-cell killing [116], triggering exosome release from HIV-1 infected cells may represent a major pathogenic determinant of this viral protein. With the enhanced understanding of the molecular mechanisms by which Nef alters T-lymphocyte responses to TCR stimulation, future investigations will undoubtedly focus on defining the patho-physiological role of these mechanisms.

### HIV-1 Nef in context of the sub-synaptic LAT concept

Studying the effects of HIV-1 Nef on TCR signaling not only provides insight into the patho-physiological mechanism of the viral protein but also contributes to our understanding of the general principles of TCR signal transduction. According to an emerging concept of TCR signaling, most of the LAT clusters at the plasma membrane are inactive at steady state and do not contribute significantly to signaling upon T-cell activation. Rather, sub-synaptic vesicles containing LAT are recruited to the plasma membrane upon TCR engagement to facilitate microcluster-dependent signaling [30,122,123]. These LAT positive vesicles move rapidly in a random manner, making transient visits at both TCR–ZAP-70 and SLP-76 clusters. Notably, colocalization of LAT containing vesicles with TCR–ZAP-70 microclusters correlates with phosphorylation of LAT [122] suggesting that LAT positive vesicles interact closely with the PM and are phosphorylated by kinases associated with triggered TCRs [30,123]. This model provides an attractive concept for the spatio-temporal regulation of LAT’s interactions with the TCR machinery [30] but has remained controversial. The recent mechanistic studies on how HIV-1 Nef usurps TCR signaling are in line with an important role of sub-synaptic LAT in TCR signaling [100,110]. Notably, Nef-expressing cells fail to generate functional p-LAT microclusters despite the presence of LAT containing microcluster at the IS. Conceivably, the pronounced intracellular accumulation of LAT induced by the viral protein prevents recruitment of LAT to the PM and thus functionalization of LAT microclusters post TCR stimulation [110]. This intracellularly accumulated LAT population in Nef expressing cells likely represents a sub-synaptic LAT vesicle pool that is prevented from translocating to TCR activation sites. In this scenario, interference with translocation of sub-synaptic LAT would be one mechanism by which Nef disrupts TCR signaling downstream of LAT. However, this disruption does not only depend on Nef’s ability to target LAT containing vesicles to this yet to be defined intracellular compartment, but also requires Nef to interfere with TCR-induced actin dynamics. This predicts that also in physiological T-cell signaling, dynamic actin remodeling may be critical for translocation of LAT positive vesicles to TCR activation sites, allowing phosphorylation of the adaptor protein and subsequent downstream signaling events.

### The LAT-SLP-76 module as target for pathogens other than HIV-1

Emphasizing the central role of TCR signaling for pathogen-host interactions, targeted modulation of LAT-SLP-76 adaptors is also employed by other proteins encoded by viral and non-viral pathogens. One example is the p12 protein of human T-cell leukemia/lymphoma virus type 1 (HTLV-1) that inhibits the phosphorylation of LAT post TCR stimulation [124]. It is believed that by decreasing T-cell responsiveness to TCR stimulation, p12 selectively curtails viral expression to minimize immune recognition of infected CD4^+^ T-cells and impair the function of infected cytotoxic CD8^+^ T-cells, thereby providing a favorable environment for viral persistence in the infected host. Similar strategies were also identified for the gram-negative bacterium *Yersinia,* whose virulence factor YopH specifically limits phosphorylation of LAT and SLP-76 to inhibit T-cell activation and thus alter T-cell mediated immune responses [125]. Finally, the T-lymphotropic tumor virus herpesvirus saimiri encodes for a “viral version” of LAT that triggers T-cell activation in infected cells by partially substituting for functions of cellular LAT [126]. Taken together, modulation of LAT-SLP-76 adaptors thus emerges as a strategy frequently used by various pathogens to adapt T-cell responses to their specific needs.

### Model of Nef action in TCR signaling

Together a scenario emerges in which Nef tailors TCR responses by relocalizing TCR proximal signaling from the PM to the RE/TGN (Figure 2). This is achieved by modulating vesicular transport routes that govern the transport of essential TCR proximal machinery such as Lck and LAT to the PM and by disrupting TCR-induced actin remodeling events critical for the spatio-temporal coordination of TCR proximal signaling machinery. Besides reducing the concentration of these components at plasma membrane sites of TCR engagement, these alterations also result in an enrichment of signaling competent TCR machinery at the RE/TGN. Still responsive to TCR engagement at the PM via the activity of Fyn, this intracellular pool triggers selective downstream pathways including activation of Erk. Presumably, many other TCR downstream effectors will not be induced in this configuration, suggesting that Nef tailors the response to TCR stimulation from a broad cascade to a specific and targeted activation of the RAS-Erk pathway. Consistent with the large body of existing literature, Nef thus can both activate and inhibit select aspects of TCR signaling and which effect predominates is likely dictated by the precise activation state of the T-lymphocyte used [88].

## Concluding remarks

Which effects HIV-1 Nef exerts on TCR signaling has remained a controversial issue with many reports describing either activating or inhibitory effects of the viral protein on select aspects of TCR signaling. Emerging evidence allows integrating these seemingly contradictory findings into a unifying model. In this scenario, Nef disrupts important TCR proximal events and simultaneously sensitizes specific downstream aspects of compartmentalized TCR signaling. This strategy allows Nef to optimize HIV-1 replication in CD4^+^ T-lymphocytes, but may also facilitate additional processes such as cell-cell communication that exert indirect effects on virus spread and/or pathogenesis. The fact that important human pathogens such as HIV-1 or HTLV-I target TCR signaling underscores the central role of this pathway for host cell interactions of T-cell tropic pathogens. The responsible pathogen factors represent valuable tools for dissecting the molecular basis of TCR signaling.

## Abbreviations

dNTP: Deoxyribonucleotide triphosphate; HIV: Human Immunodeficiency virus; IS: Immunological synapse; ITAM: Immunoreceptor tyrosine-based activation motif; LAT: Linker of Activated T-cells; Lck: Lymphocyte-specific protein tyrosine kinase; MAPK: Mitogen activated protein kinase; MCs: Microclusters; MHC: Major Histocompatibility Complex; NAKC: Nef-associated kinase complex; PM: Plasma membrane; RE: Recycling endosome; RT: Reverse transcription; SIV: Simian immunodeficiency virus; SLP-76: Src homology 2 (SH2) domain–containing leukocyte phosphoprotein of 76 kD; TCR: T-cell antigen receptor; TGN: Trans-Golgi network; ZAP-70: Zeta-chain-associated protein kinase 70.

## Competing interests

The authors declare that they have no competing interests.

## Authors’ contributions

LA generated the figures and OTF wrote manuscript together with LA. Both authors read and approved the final manuscript.
